# Quantitative Study of Using Piloti for Passive Climate Adaptability in a Hot-Summer and Cold-Winter City in China

**DOI:** 10.3390/ijerph15102202

**Published:** 2018-10-09

**Authors:** Zeng Zhou, Qinli Deng, Guang Yang, Yaolin Lin

**Affiliations:** 1 School of Urban Design, Wuhan University, Wuhan 430070, China; haomaoz@whu.edu.cn; 2 School of Civil Engineering and Architecture, Wuhan University of Technology, Wuhan 430070, China; yaolinlin@gmail.com; 3 College of Engineering and Architecture, Liaoning Technical University, Fuxin 123000, China; yangguang_tm@lntu.edu.cn

**Keywords:** climate adaptation, outdoor thermal environment, hot-summer and cold-winter, Piloti

## Abstract

There has been an insufficient study of passive climate adaptability that considers both the summer and winter season for the outdoor thermal environment of hot-summer and cold-winter cities. In this study, we performed a quantitative simulation to research the passive climate adaptability of a residential area, considering piloti as the main method for climate adaptation in a hot-summer and cold-winter city in China. Numerical simulations were performed with a coupled simulation method of convection, radiation, and conduction. A cubic non-linear k–ε model proposed by Craft et al. was selected as the turbulence model and three-dimensional multi-reflections of shortwave and longwave radiations were considered in the radiation simulation. Through the simulation, we found that setting the piloti at the two ends of the building was the optimal piloti arrangement for climate adaptation. Then the relationship between the piloti ratio (0%, 20%, 40%, 60%, and 80%) and the outdoor thermal environment was studied. It could be concluded that with the increasing piloti ratio, the wind velocity increased, the mean radiant temperature (MRT) decreased slightly, and the average standard effective temperature (SET*) decreased to 3.6 °C in summer, while in winter, with the increasing piloti ratio, the wind velocity, MRT, and SET* changed slightly. The wind environment significantly affected the SET* value, and the piloti ratio should be between 12% and 38% to avoid wind-induced discomfort.

## 1. Introduction

With the aggravation of global warming and the expansion of urbanization, serious environmental problems such as air pollution, large anthropogenic heat release, and urban heat island are severer day by day [1,2,3,4]. The Urban Heat Island has caused outdoor thermal environments in residential areas to become worse [5]. Body discomfort and heat stroke rates have increased significantly because of the deterioration of the indoor and outdoor thermal environment [6,7]. Since people feel hotter in the summer, air conditioning is used more frequently and for a longer time; then, the exhaust gas from air conditioning makes the outdoor thermal environment in residential areas worse [8,9]. Therefore, avoiding the vicious cycle of the deterioration of the thermal environment, energy consumption, and further deterioration of the thermal environment’ has become an urgent problem in urban development.

China has rapidly increasing energy needs; building energy consumption accounts for approximately one-third of the total social consumption, and this energy consumption is expected to continue to grow [10]. Passive climate adaptability architectural designs have low energy consumptions, zero energy consumptions, and even negative energy consumptions [11], so it has attracted more research attention. Many studies have focused on optimizing the outdoor thermal environment such as greenery, water features, and pavement materials [12,13,14,15,16,17,18]; other studies have focused on optimizing buildings, such as the layout, height, and density [19,20,21,22,23,24,25,26]. However, piloti, a ubiquitous architectural form in the hot summer areas of southern China, have not been sufficiently considered in the quantitative studies of passive climate adaptability design.

Setting optimal piloti is a good way to adapt to the Urban Heat Island. Piloti can form shaded space and enhance the surrounding wind speed, but it does not raise the air humidity like trees, so that the effect of the piloti on mitigating the outdoor human thermal comfort can be highly anticipated [27,28]. Chen et al. studied the influence of the arrangement of piloti on the outdoor wind environment in Shenzhen, China [28]. They found that the optimal piloti arrangement could highly improve the outdoor thermal environment in summer. Li clarified the relationship between the piloti arrangement and the velocity ratio under different wind directions, but this research did not consider the influence of the surrounding buildings and no thermal comfort index was included [29]. Xi researched the influence of the piloti ratio on the outdoor thermal environment of residential blocks in Guangzhou, China, in the summer [30]. It was found that the outdoor thermal environment is improved with the increase of the piloti ratio, however, Xi set piloti in all the buildings studied, but this extreme situation rarely occurs outside of the research environment. 

Chen et al. simulated the effects of piloti on the outdoor thermal environment of a campus building in Guangzhou [31]. Different heights of piloti and proportions of piloti were researched. However, during the hottest months in summer the university is under the summer vacation. Zhao et al. studied the effects of piloti and non-piloti forms on the outdoor wind environment using the teachers’ apartments of Wenzhou University as an example [32]. They found that wind environment at piloti significantly improved; the flow field within the district tends to be smooth with no significant turbulence zone, and the piloti can beneficially improve the air circulation and reduce the air pollution retention. Zhou et al. researched the effects of piloti on the outdoor thermal environment of a residential area in Wuhan in summer [33]. They found that the pedestrian wind environment and the outdoor thermal environment improved with the increase of the piloti ratio. These studies show that the piloti can effectively improve the regional thermal environment in summer. It could be known from the above research that piloti are a really good element to improve outdoor thermal environments. However, there are some limitations in these studies: (i) Most of the researches only focus on the summer period; (ii) Many studies ignore the influence of the surrounding buildings without piloti. (iii) Some studies only discuss the relationship between the wind velocity and piloti, but do not pay attention to mean radiant temperature (MRT) and standard effective temperature (SET*).

In this study, (i) we conducted a quantitative simulation of passive climate adaptability in both summer and water for a residential area; (ii) we used piloti as the main method to improve the outdoor thermal environment and the influence of surrounding buildings without piloti is also considered; (iii) Outdoor thermal environment including wind velocity, surface temperature, MRT and SET* was studied. Our main objects were to propose an arrangement of piloti for climate adaptation and to determine the optimal piloti ratio for residential areas in a hot-summer and cold-winter city.

## 2. Analysis Outline

### 2.1. The City of Wuhan

**Figure 1:**
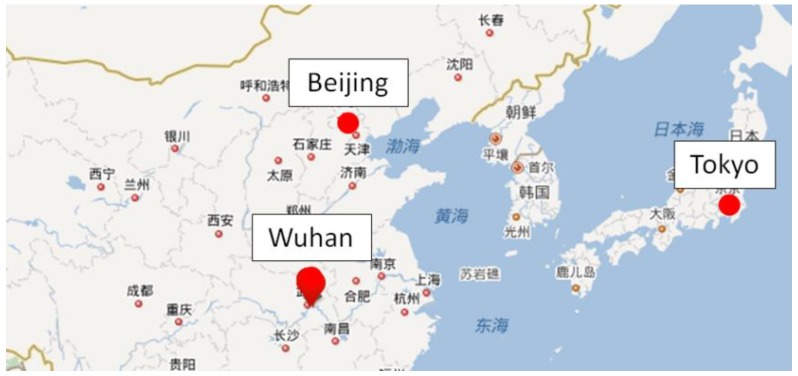
Location of Wuhan.

As shown in Figure 1, Wuhan is located in central China, and it is the core city of the Yangtze River economic belt. Wuhan lies east of the Jianghan Plain, at the intersection of the middle reaches of the Yangtze River and the Han River, and there are more than 100 lakes that the humidity in Wuhan is high all year round. Wuhan is a typical hot-summer and cold-winter city in China. It is hot and humid in summer, moreover cold and humid in winter; therefore, during most of the year, people in Wuhan are under conditions of thermal discomfort. The highest temperature could be almost 39 °C, and the lowest temperature could be less than −3 °C.

### 2.2. Prediction Method for the Outdoor Thermal Environment

A coupled approach of convection, radiation, and conduction for the three-dimensional outdoor thermal environment was first initiated by Yoshida et al. based on a self-edited program [34]. They compared the results of simulations with field measurements from a northern part of Tokyo. They used two domains, a large one and a small one; moreover, the large one contained the small one. It was a two-stage coupled analysis. Firstly, a computational fluid dynamics (CFD) analysis was performed in the large area, and measurement values were used as boundary values. They selected a k–ε revised turbulence model and obtained small area’s boundary values belonging to the large area. They conducted the CFD analysis in the small area using outcomes in the first step and air temperature and humidity from on-site observations. Finally, they found that results from the numerical analysis agreed with the on-site observations.

For this study, we conducted a non-isothermal CFD analysis using data coming from the local meteorological bureau containing wind velocity, prevailing wind direction, and air temperature, and observed data containing ground and surface temperatures. The ground and building surface temperatures were calculated in step 2 based on the unsteady state heat balance calculation, including three-dimensional radiation and one-dimensional conduction calculations. Based on the results in these two steps, we once again conducted a CFD analysis to obtain more accurate results. Finally, we calculated SET*, a comprehensive index, to evaluate outdoor thermal comfort in the target area, which was in the centre of the analysis domain. Xi [31], Xuan et al. [32] and Zhou et al. [34] conducted their research using a similar prediction method.

### 2.3. Analysis Date and Time

#### 2.3.1. Simulation Date

In this study, a weather database of China [35] was used. The typical meteorological day in summer is 1 July and the typical meteorological day in winter is 2 January (Table 1).

#### 2.3.2. Simulation Time

We conducted an internet-based questionnaire survey in 2013 to determine the time of day when residents go outdoors in the residential district. More than 300 survey answers were collected. The question used in the survey is shown in Table 2.

**Figure 2:**
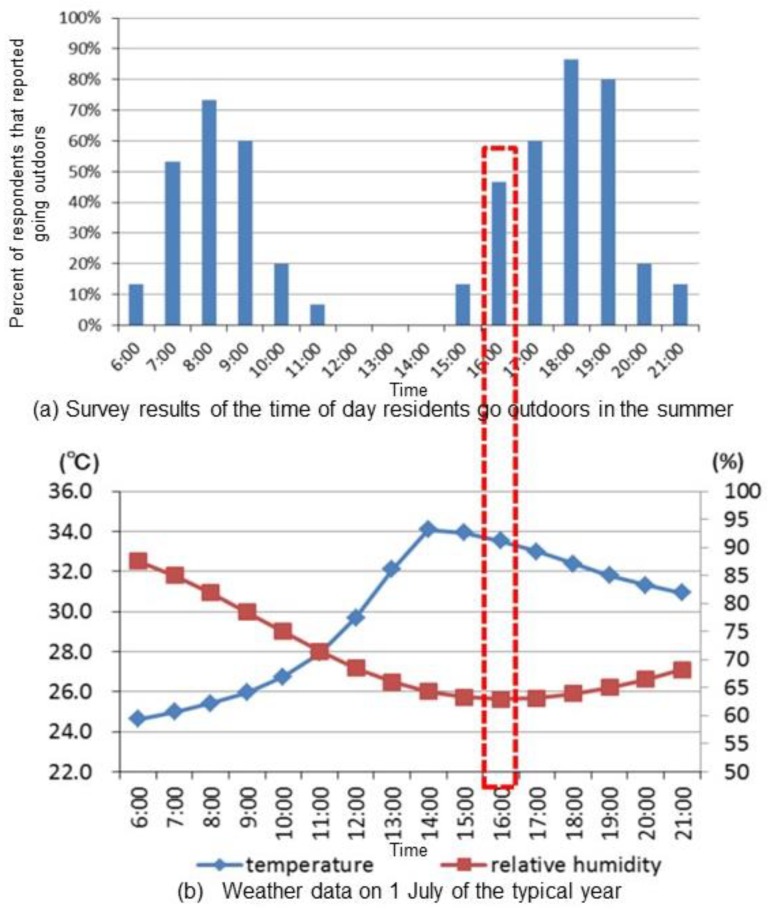
The survey results of the time of day residents go outdoors in the summer (**a**) and the weather data on the 1 July of a typical year (**b**).

Figure 2 presents the survey results and weather data for 1 July of a typical year. The simulation time was set at 16:00 in the summer because of the high percentage of people who reported going outdoors (approximately 50%) and the high temperature (approximately 34 °C).

**Figure 3:**
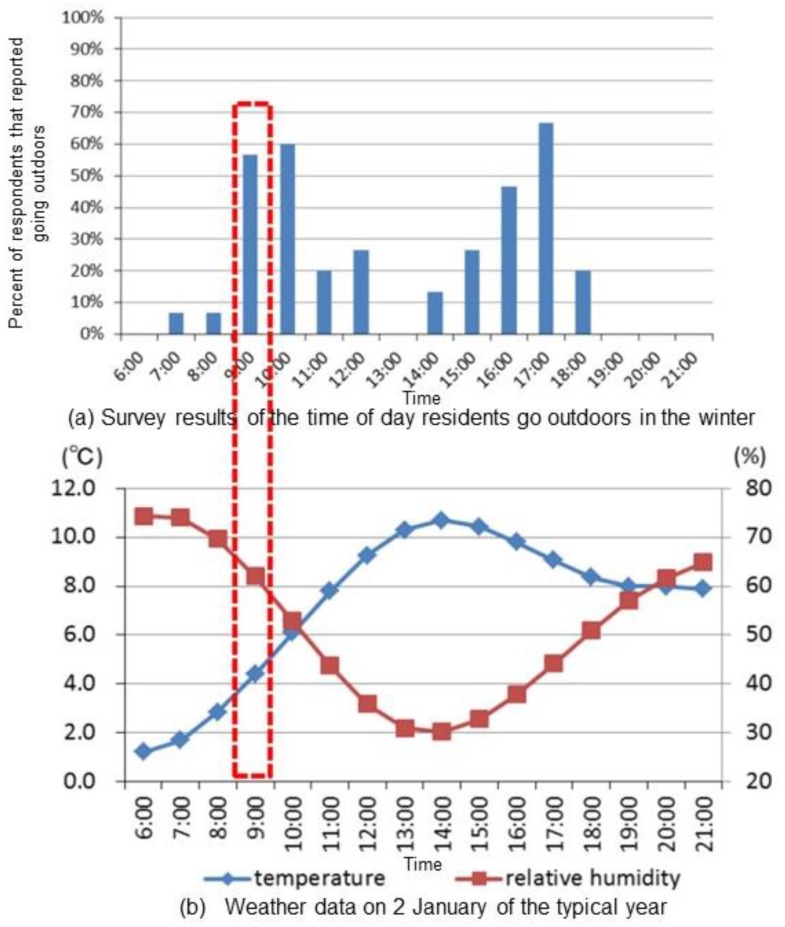
The survey results of the time of day residents go outdoors in the winter (**a**) and weather data on the 2 January of a typical year (**b**).

Figure 3 presents the survey results for winter and the weather data on 2 January of a typical year. The simulation time was set to 9:00 in the winter because of the high percentage of people who go outdoors at that time (approximately 60%) and the low air temperature (approximately 4 °C).

### 2.4. Analysis Method and Conditions

**Figure 4:**
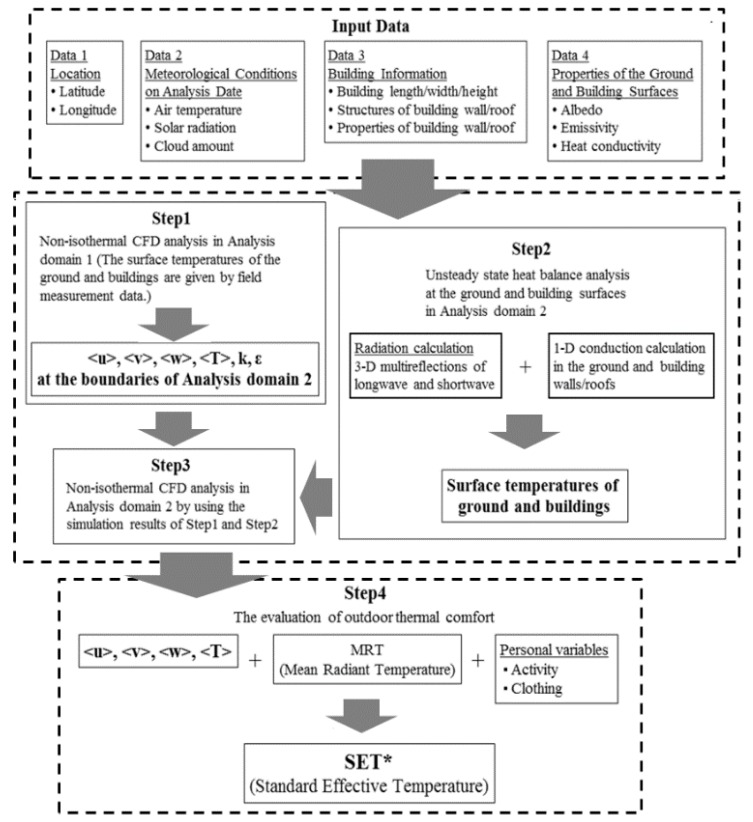
The flowchart of the prediction method for the outdoor thermal environment.

In this study, we used a numerical analysis system by integrating STAR-CD/RADX (CD adapco Group, Melville, NY USA) with additional codes. Figure 4 shows the flowchart of a coupled approach applied in this study. Our analysis was conducted in four steps. In step 1, we conducted a non-isothermal CFD analysis. We used meteorological conditions (air temperature, wind velocity, and prevailing wind direction) and ground and building surface temperatures observed in the environment as the initial and analysis conditions (Table 3).

In step 2, we conducted an unsteady state heat balance analysis for 16:00 on 1 July obtained from the ground and building surface temperatures. We included three-dimensional radiation and one-dimensional conduction calculations in this process (Table 4). In step 3, we conducted a non-isothermal CFD analysis on account of the results of step 1 and step 2. The analysis conditions for step 3 are shown in Table 5. In step 4, we evaluated the thermal comfort for the study area with outcomes from step 3 (wind velocity, air temperature, humidity, and the mean radiant temperature (MRT)) and personal variables (activity and clothing). Table 6 lists the structures of the ground and buildings (wall and roof) and the corresponding thermal properties. Table 7 lists the surface properties of the ground, grass, building wall, and roof.

### 2.5. The Accuracy of the Analysis Method

#### 2.5.1. Turbulence Model for Computational Fluid Dynamics (CFD) Analysis

A cubic non-linear k–ε model proposed by Craft et al. [36] is selected as the turbulence model for the CFD analysis. This turbulence model was originally developed to capture turbulence anisotropy by proposing a cubic relation between stress and strain, which significantly improves the predictions of three-dimensional fluid flow and heat transfer not only in and around a single object [36,37,38], but also around several objects [39]. This model has been successfully and widely used in mechanical engineering because of its high prediction accuracy. Its application in the field of wind climate around buildings is tested for the first time by our group. Specifically, its performance is compared with two other turbulence models (the standard k–ε model and the quadratic non-linear k–ε model proposed by Lien et al. [40]) and shows the best agreement with a wind tunnel experiment from the AIJ benchmark cases [41,42]. 

Li et al. [43] compared 15 kinds of turbulence models for outdoor wind environment numerical simulation using STAR-CD with the wind tunnel test provide by AIJ [41,44] and the results show that the Suga cubic non-linear k–ε model is of high precision for wind field solution.

#### 2.5.2. Heat Transfer Analysis

To calculate urban surface temperatures, all the surfaces in the computational domain are divided into small surfaces. For each small surface, solar radiation, sky radiation, longwave radiation between it and other surfaces, convective heat transfer and latent heat transfer between it and the ambient air, and conduction heat transfer through it are considered. The shape factor is calculated using the Monte Carlo method [45,46] and the radiative heat transfer is calculated using Gebhart’s absorption factor [47]. Given that the values of Gebhart’s absorption factor are different because of the different absorptivities under shortwave and longwave radiations, they are calculated separately for each surface. According to previous studies [28,48,49], urban surface temperatures are well reproduced by considering the heat transfers mentioned above.

### 2.6. Analysis Model and Cases

**Figure 5:**
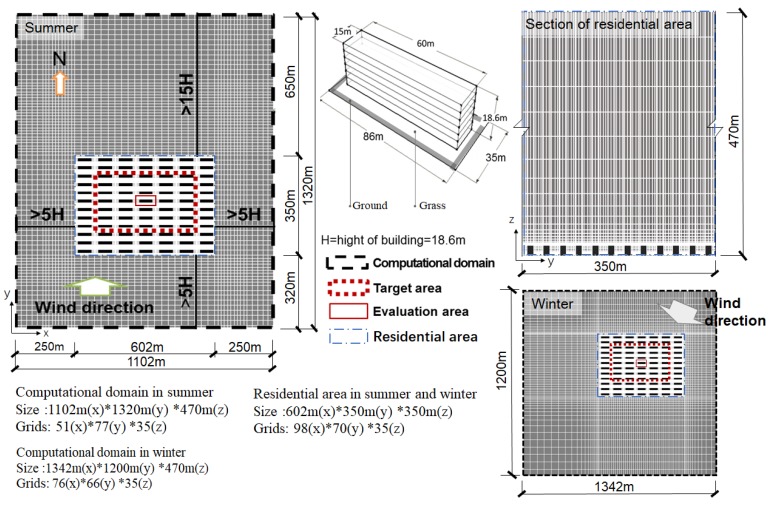
The analysis model used in this study.

The analysis model used in this study is shown in Figure 5. We determined the size of the computational domain based on the Architectural Institute of Japan Guidelines [50]. The size of computational domain in summer was 1102 m (x) * 1320 m (y) * 470 m (z), and the size in winter was 1342 m (x) * 1200 m (y) * 470 m (z). The grids of the computational domain in summer and in winter were 51(x) * 77(y) * 35(z) and 76(x) * 66(y) * 35(z), respectively. The sizes and grids of the residential area in summer and winter were same. We used six-floor residential buildings as the research object as they are the most common buildings in the study area. The density of the buildings was approximately 30% and the height of the first floor was 3.6 m; the height of other floors was 3 m.

**Figure 6:**
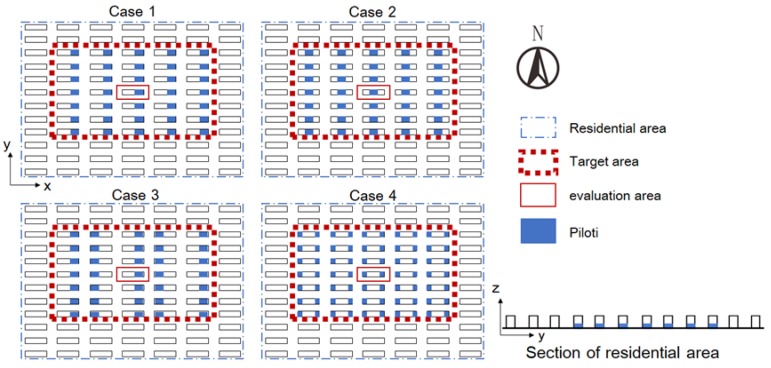
The piloti arrangements analysed for the study area.

As seen in Figure 6, four different piloti arrangements were considered in the study area to determine the optimal arrangement (Table 8). Then, we investigated the relationship between the piloti ratio and the wind environment based on the optimal piloti arrangement.

## 3. Results

### 3.1. The Optimal Piloti Arrangement

#### 3.1.1. Surface Temperature

**Figure 7:**
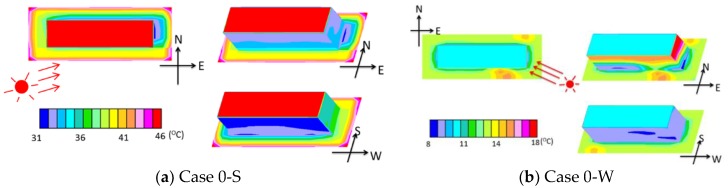
The surface temperatures for Case 0-S and Case 0-W.

The surface temperatures of Case 0-S are presented in Figure 7a. For the building, the temperature of the roof surface was the highest, reaching 46 °C, followed by the west wall (approximately 37 °C). The temperature of the north wall was the lowest (approximately 32 °C). The direction of sunshine in the summer case is also shown in the figure.

The surface temperatures of Case 0-W are presented in Figure 7b. In this case, the sunshine reached the 6th or 5th floor. For the building, the east wall had the highest temperature (approximately 18 °C). Most of the ground was in shadow. The temperature of the ground was approximately 32 °C. The wind environment was more important than the thermal environment in the winter case because most of the building and ground were in shadow. Therefore, to save time, the MRTs of the winter cases were not calculated.

**Figure 8:**
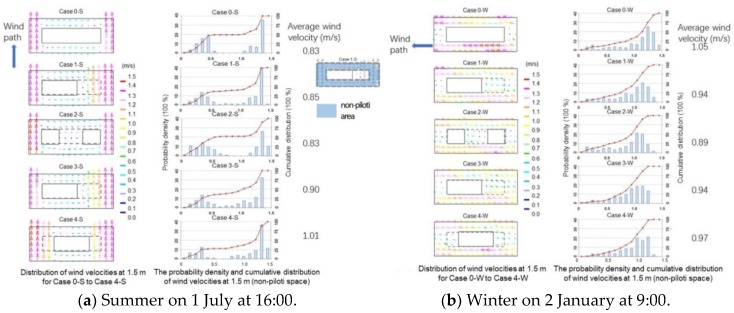
The wind velocity results.

Figure 8a presents the distributions of wind velocity at 1.5 m for Case 0-S to Case 4-S, and it shows the probability density and cumulative distribution of wind velocity for Case 0-S to Case 4-S. When the piloti ratio was 0 (Case 0-S) or the piloti was in the middle of the building (Case 2-S), the portion of wind velocity less than 0.5 m/s occupied about half of the cumulative distribution. When the piloti was at the two ends of the building (Case 4-S), the wind velocities greater than 0.5 m/s were over 75% of the cumulative distribution. Case 4-S significantly improved the wind environment. Therefore, we concluded that the piloti at the two ends of the building was the optimal piloti arrangement. The left part of Figure 8b shows the distributions of wind velocities at 1.5 m for Case 0-W to Case 4-W. The wind path formed along the east to west direction. After adding the piloti space, the wind path weakened. The right part of Figure 8b presents the probability density and cumulative distribution of the wind velocities at 1.5 m of non-piloti space. After adding piloti, the maximum wind velocity decreased. From Case 1-W to Case 4-W, all the average wind velocities were lower than Case 0.

#### 3.1.2. MRT and SET*

**Figure 9:**
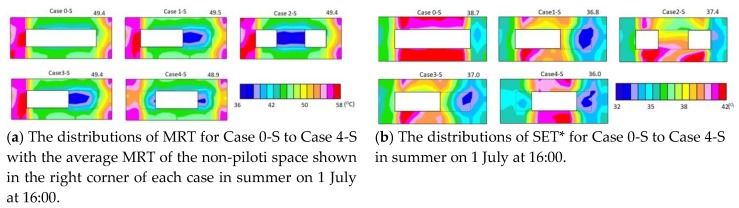
The MRT and SET* results for the piloti arrangements in summer.

Figure 9a presents the distributions of the MRT for Case 0-S to Case 4-S. The west wall had the most significant effect on its surrounding MRT. The surface temperature of the west wall on the first-floor in Case 4-S was low, so the MRT surrounding the west wall was lower than other cases. The shaded space under the piloti also decreased the MRT value. Therefore, Case 4-S had the lowest MRT.

The distributions of SET* for Case 0-S to Case 4-S are shown in Figure 9b. The MRT was significantly affected by wind velocity. The wind velocity was very weak besides the south and north sides of the building, and although the MRT was very high, the SET* was also very high. The highest SET*, adjacent to the south side of the building, reached approximately 42 °C. In Case 2-S, although the MRT under the piloti was very low, the SET* was very high (approximately 39 °C) because the wind velocity under the piloti in Case 2-S was very weak. In other cases, the lowest SET* was under the piloti space (approximately 32 °C). The SET* along the wind path was low because of the high wind velocity. The average SET* of the non-piloti space from Case0-S to Case4-S were 38.7 °C, 36.8 °C, 37.4 °C, 37 °C, and 36 °C, respectively. The piloti arrangement of Case4-S decreased the SET by 2.7 degrees and shows the best passive effect in summer. 

This study gave priority to the improvement of a thermal environment in summer because (i) Wuhan is famous for its hot and humid summer in China, and the climate problem is more serious in summer and because (ii) in cold-winter days people can easily improve their outdoor thermal comfort by clothing, warm paste and exercise, while in hot-summer days, the effect of such methods is very limited. In summer, the piloti arrangement of case 4 shows the best effect in wind environment (Figure 8a) and SET*(Figure 9b), and in winter, the arrangement of case 4 can decrease the average wind velocity although it was not the best (Figure 8b). However, the optimal piloti arrangement is determined by the thermal environment in summer as the objective of the design is to solve the heat stress problem. Although there could be some improvements on the MRT and SET* in winter by altering the piloti arrangement, it will have no impact on the conclusion in this paper.

### 3.2. Relationships between the Piloti Ratio and the Outdoor Thermal Environment

#### 3.2.1. Wind Velocity

**Figure 10:**
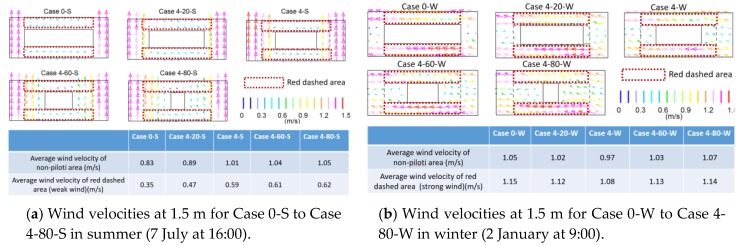
The wind velocity results for the piloti arrangements.

The upper part of Figure 10a shows the distributions of wind velocities at 1.5 m for Case 0-S to Case 4-80-S. The wind path formed along a south to north direction. With an increasing piloti ratio, the wind path increased. The wind velocities beside the south and north side of the building were very weak, and the enlarged wind path improved the wind velocity of this area. The lower part of Figure 10a lists the average wind velocities of different summer cases. The wind environment was improved with the increasing piloti ratio in the summer. The wind velocity in the red dashed areas (weak wind areas) changed significantly.

The upper part of Figure 10b shows the distributions of wind velocities at 1.5 m for Case 0-W to Case 4-80-W. The wind path formed in the east to west direction. With the increasing piloti ratio, the wind path weakened. The lower part of Figure 10b lists the average wind velocities of different cases in winter. The wind environment changed slightly in winter with the increasing piloti ratio. With the increasing piloti ratio, the average wind velocity first decreased and then increased; with the increasing piloti ratio, the wind path weakened and the wind velocity decreased. On the first floor, the building density decreased and the wind velocity increased.

The average wind velocity of one month was much higher than the wind velocity for the analysis time. To avoid wind-induced discomfort, it is better to consider the average wind velocity for one month. Based on the input data, we calculated the wind velocity at a height of 1.5 m (0.78 m/s in the summer and 1.24 m/s in the winter). According to the definition of the wind velocity ratio, the relationship between the piloti ratio and the average wind velocity ratio can be represented as

(1) $$R_{i}=\langle u_{i}\rangle /\langle u_{i0}\rangle$$

*R_i_* is the wind velocity ratio.

〈*u_i_*〉 is the wind speed at point *i* (m/s) when buildings exist.

〈*u_i_*_0_〉 is the wind speed at point *i* (m/s) when buildings do not exist, usually the same as the inflow value at the same height.

**Figure 11:**
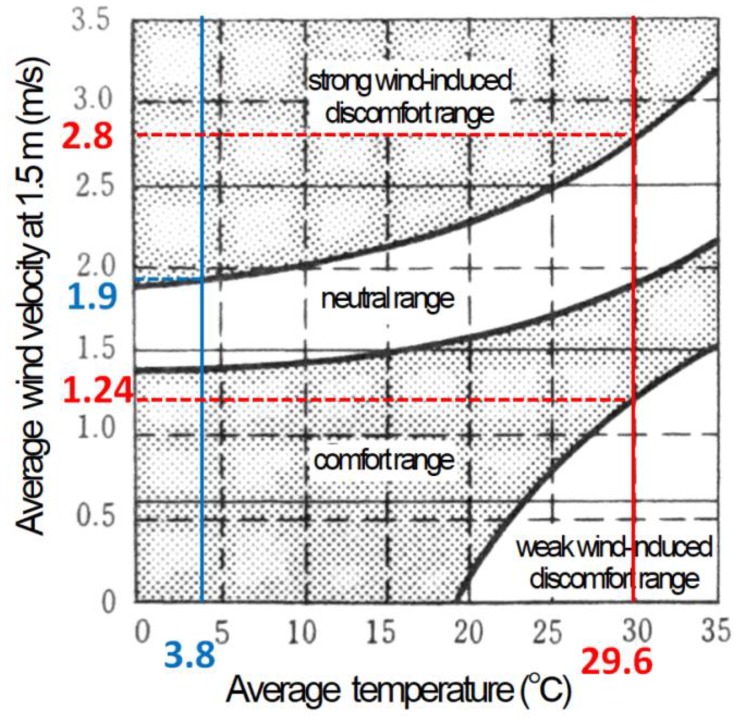
The criteria for assessing the wind-induced discomfort considering the temperature effect.

According to a weather database of China [35], the average wind velocity in July (the hottest month) is 3.6 m/s at the height of 10 m, and the average wind velocity in January (the coldest month) is 2.4 m/s at the height of 10 m. According to the input data (ground roughness α = 0.25), we calculated that the average wind velocities at the height of 1.5 m in summer and winter are 2.24 m/s and 1.49 m/s, respectively. The main part of Figure 11 was generated from the data in Table 9. Table 10 presents the relationship between the piloti ratio and the average wind velocity of the non-piloti area and the red dashed area in summer and in winter. 

**Figure 12:**
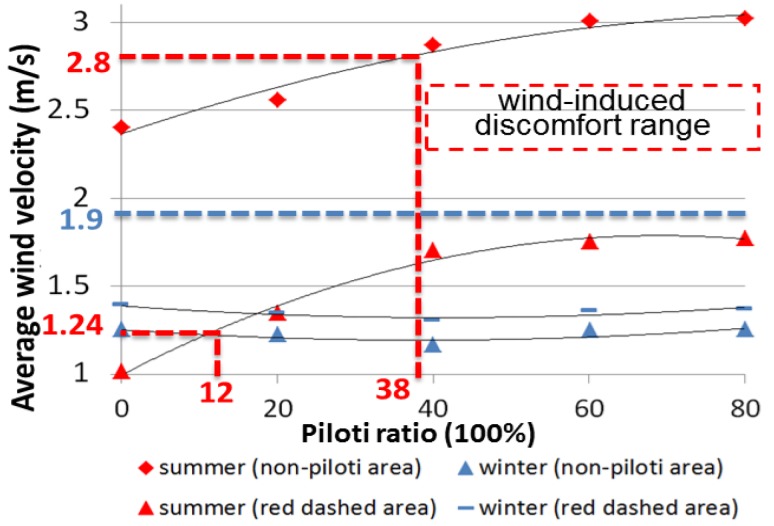
The relationship between the piloti ratio and the average wind velocity of non-piloti areas.

The main content of Figure 11 was developed from Murakami’s research [51]. According to a weather database of China [35], the average temperature in July (the hottest month) is 29.6 °C and the average temperature in January (the coldest month) is 3.8 °C. As seen in Figure 11, if the wind velocity is stronger than 2.8 m/s or weaker than 1.24 m/s, it will cause wind-induced discomfort in summer; if the wind velocity is stronger than 1.9 m/s, it will cause wind-induced discomfort in winter. Figure 12 shows that the piloti ratio should be between 12% and 38% to avoid wind-induced discomfort.

#### 3.2.2. MRT

**Figure 13:**
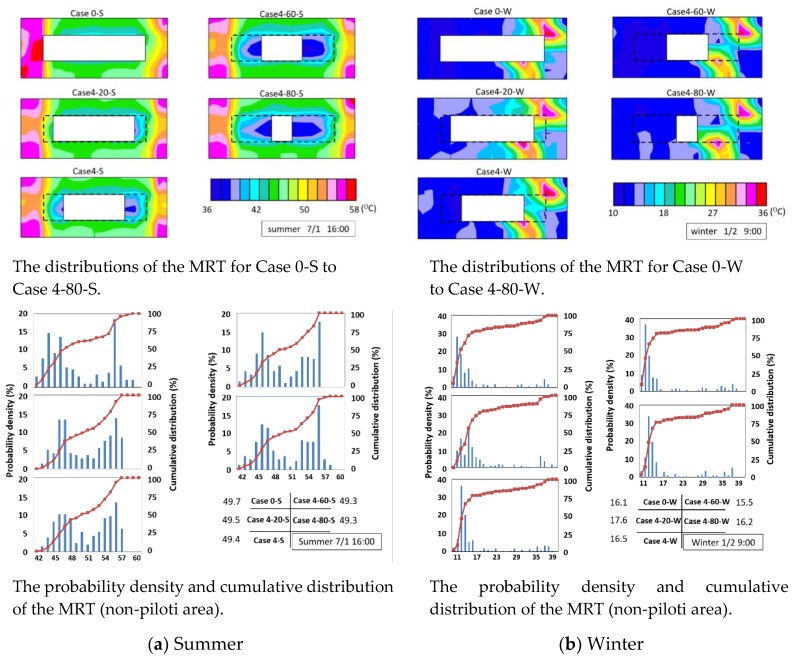
The simulation results of the MRT.

The distributions of MRT for Case 0-S to Case 4-80-S are displayed in the upper part of Figure 13a. With the increasing piloti ratio, the MRT decreased. A shaded space formed under the piloti. In Case 0-S, the temperature of the west wall on the first floor was high, so the MRT beside the west wall was very high. After adding piloti, the west wall on the first floor was under the piloti, so the temperature of the west wall in the first floor was less, and the MRT beside the west wall was low because of the shaded space under the piloti. The lower part of Figure 13a shows the probability density and cumulative distribution of the MRT of the non-piloti area.

The distributions of the MRT for Case 0-W to Case 4-80-W are displayed in the upper part of Figure 13b. In the winter, the MRT changed slightly after adding piloti. The lower part of Figure 13b shows the probability density and cumulative distribution of MRT (non-piloti area) in the winter. Although the average MRT changed slightly with the increasing piloti ratio, the average MRT first increased, then decreased, and then increased again. The area that received direct sunlight increased with the increasing piloti ratio and this caused the MRT to increase. It was difficult to receive diffuse radiation from the sky under the west piloti, so its MRT was lower than the surrounding space, and this decreased the surrounding MRT.

#### 3.2.3. SET*

**Figure 14:**
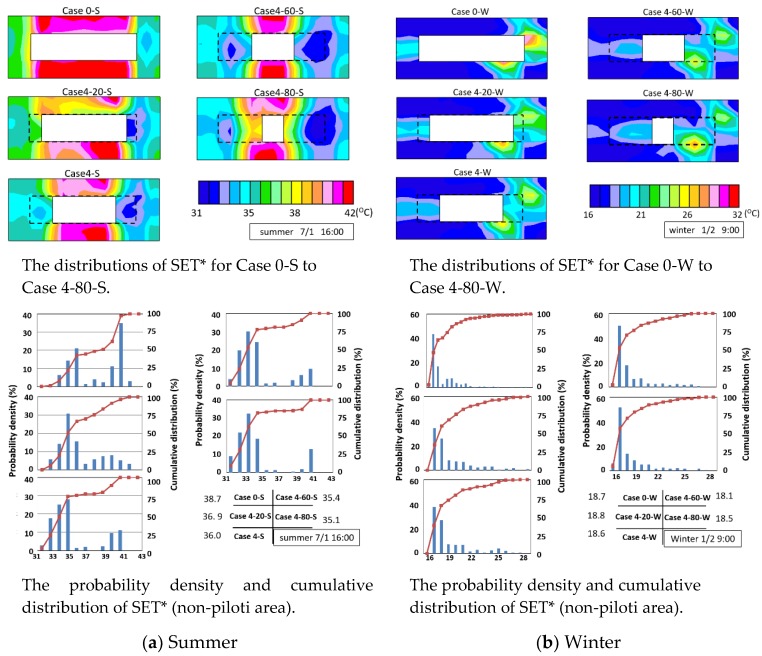
The simulation results of SET*.

The upper part of Figure 14a shows the distributions of SET* in the summer cases. Piloti can provide shaded places and this can rapidly decrease the SET*. The SET* was very high beside the south and north sides of the building because the wind velocity was very weak. The SET* was lower beside the east and west sides of the building because of the wind path. The lower part of Figure 14a presents the probability density and cumulative distribution of SET* (non-piloti area) for the summer cases. The minimum SET* value was approximately 31 °C, and the maximum SET* value was approximately 42 °C.

The upper part of Figure 14b presents the distributions of SET* for Case 0-W to Case 4-80-W. The SET* value of the area that received direct sunshine was highest and most of the area was in shadow. The SET* beside the two ends of the building was higher than that of other areas because the wind velocity besides the two ends of the building was very weak. The SET* changed slightly after adding piloti. The lower part of Figure 14b displays the probability density and cumulative distribution of SET* of the non-piloti area for the winter cases. The minimum SET* value was approximately 16 °C, and the maximum SET* value was approximately 29 °C. With the increasing piloti ratio, the average SET* first increased, then decreased, and then increased again. This trend was likely due to the changing trends of wind velocity and the MRT. The SET* values changed slightly in winter with the increasing piloti ratio.

## 4. Discussion

In this paper, case studies of a residential area with different piloti arrangements and the ratio by numerical simulation were carried out, and the piloti showed good passive climate adaptability in a hot summer and cold winter city. The relationships between piloti variables and outdoor thermal environment (T, V, MRT, SET*) of the residential area both in winter and in summer in Wuhan is studied for the first time. Data in Table 6 can be converted to the relationship between piloti ratio and wind speed in other cities with similar climate with Wuhan, which shows a certain adaptability.

Although there are some interesting results revealed by this paper, there are also limitations. First, we only studied the most basic and common determinant that arranged the residential area, while other arrangement forms (point, enclosed) with different building shapes also accounted for a large proportion. Second, we only consider the piloti variable, however, the improvement of the thermal environment is not limited by a single piloti variable. Other passive climate adaptability elements, such as reflective materials and the green wall should also be taken into account. Third, Trees, pedestrians, and cars are omitted in the simulation. Fourth, only a typical meteorological day is considered in this study, other typical days such as the hottest day and the coldest day could also be taken into account.

In our future work, the thermal environment of residential areas under different arrangement forms (point, enclosed) will be researched.

## 5. Conclusions

We studied the outdoor thermal environment in the hottest month (July) and coldest month (January) in Wuhan. We found that setting piloti at the two ends of the building was the best piloti arrangement for climate adaptation. It improved the wind environment and thermal environment. 

Based on the optimal piloti arrangement, we studied the relationship between the piloti ratio (0%, 20%, 40%, 60%, and 80%) and the outdoor wind environment. In the summer, the wind velocity increased with the increasing piloti ratio, as the piloti could strengthen the wind path (from south to north). In the winter the wind environment changed slightly with the increasing piloti ratio because the piloti had little effect on the wind path (from east to west). The criteria for assessing wind-induced discomfort considering the temperature effects were used to evaluate the wind environment. We found that the piloti ratio should be between 12% and 38% to avoid wind-induced discomfort.

In the summer, with the increasing piloti ratio, MRT and SET* decreased. The wind path significantly affected the SET* value. MRT and SET* changed slightly with the increasing piloti ratio because most of the building and ground were in shadow in the winter cases.

## Figures and Tables

**Table 1:** The typical meteorological day in winter and summer for Wuhan.

Average Data	2 January	1 July
Temperature (°C)	5.982	28.640
Relative humidity (%)	54.167	75.708
Solar radiation (W/m^2^)	97.685	188.285
Wind velocity (m/s)	1.250	1.250

**Table 2:** The question used in the survey.

Q: During summer/winter in Wuhan, which time periods do you always go to an outdoor place in the residential district? Please mark “○” in the box under the time period. 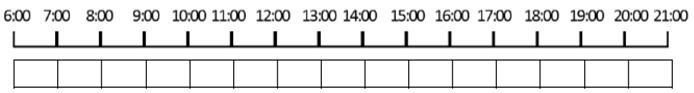

**Table 3:** The step 1 analysis conditions.

Date and Time	Summer: 16:00, 1 July Winter: 9:00, 2 January
**Calculation state**	Steady state
**Turbulence model**	Suga cubic non-linear k–ε model
**Inflow**	Wind direction: south/north-northeast. Air temperature: 33.5 °C (summer), 4.4 °C (winter) <*u*>: *U*(*z*) = <*U_s_*>(*z*/*z_s_*)*^α^* *A* = 0.25, *z_s_* = 10 m, <*U_s_*> = 1.25 m/s <*k*>: $k\left(z\right)={\left(I\left(z\right)\langle u\left(z\right)\rangle \right)}^{2}$, $I\left(z\right)=0.1\left(\frac{z}{z_{G}}\right)\left(-\alpha -0.05\right)$, $z_{G}=470m$, α=0.3 *ε*: $\varepsilon \left(z\right)=C_{\mu }^{\frac{1}{2}}k\left(z\right)\frac{\langle U_{s}\rangle }{Z_{s}}\alpha {\left(\frac{z}{z_{S}}\right)}^{\left(\alpha -1\right)}$. *C_μ_* = 0.09
**Outflow**	<*u*>, <*v*>, <*w*>, *k*, *ε*, zero gradient, <*w*> = 0, *T*: adiabatic
**Lateral and upper surfaces**	<*u*>, <*v*>, *k*, *ε*: zero gradient, <*w*> = 0
**Ground and building surfaces**	Summer: 48 °C (ground surface), 39 °C (building surfaces) Winter: 10 °C (ground surface), 8 °C (building surfaces)
**Advection term surfaces**	<*u*>, <*v*>, <*w*>, *k*, *ε*, *T*: MARS
**Coupling algorithm**	SIMPLE

**Table 4:** The step 2 analysis conditions.

Date and Time	0:00–24:00 on 1 July 9:00, 1 January–9:00, 2 January
Calculation state	Unsteady state
Temperature	The daily temperature change mode on 1 July (summer), 1–2 January (winter)
Convective heat transfer coefficient	Indoor: 5 W/m^2^·K Outdoor: 12 W/m^2^·K

**Table 5:** The step 3 analysis conditions.

Date and Time	Summer: 16:00, 1 July Winter: 9:00, 2 January
Calculation state	Steady state
Turbulence model	Suga cubic non-linear *k*–*ε* model
Inflow boundary, lateral and upper surfaces	<*u*>, <*v*>, <*w*>, *k*, *ε*, *T*: from step 1
Outflow	<*u*>, <*v*>, <*w*>, *k*, *ε*, *T*: zero gradient
Ground and building surfaces	Logarithmic law Tsurface: from step 2
Scheme for advection term	<*u*>, <*v*>, <*w*>, *k*, *ε*, *T*: MARS
Coupling algorithm	SIMPLE

**Table 6:** The structures and thermal properties of the ground, wall, and roof.

	Structure	No. of Layers	Material for Each Layer	Thickness (mm)	Thermal Conductivity (W/(m·K))	Specific Heat per Unit Volume (kJ/m^3^·K))
Ground	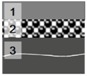	1	Concrete	100	1.28	1900
		2	Gravel	100	0.62	1500
		3	Soil	300	1.50	3100
Wall	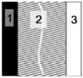	1	Exterior mortar	25	0.93	1890
		2	Aerated concrete	200	0.22	735
		3	Interior mortar	25	0.87	1785
Roof	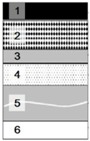	1	Exterior mortar	25	0.93	1890
		2	Fine-stone concrete	40	1.51	2116
		3	Cement mortar	20	0.93	1890
		4	Insulation layer	30	0.036	41.4
		5	Reinforced concrete	100	1.74	2300
		6	Interior mortar	25	0.87	1785

**Table 7:** The surface properties.

	Long Wave Emissivity	Albedo
Ground	0.90	0.20
Grass	0.95	0.15
Building wall and roof	0.95	0.30

**Table 8:** The details of the pilotis arrangements analysed in the study area.

Case	Analysis Date and Time	Inflow Wind Velocity (m/s)	Piloti Arrangement	Politis Ratio (%)
Case 0-S	1/7 16:00	1.25 (at 10 m, prevailing wind direction: south)	no pilotis	0
Case 1-S			at the east end of the building	40
Case 2-S			in the middle of the building	40
Case 3-S			at one end of the building	40
Case 4-S			at the two ends of the building	40
Case 4-20-S				20
Case 4-60-S				60
Case 4-80-S				80
Case 0-W	2/1 9:00	2.0 (at 10 m, prevailing wind direction: NNE)	no pilotis	0
Case 1-W			at the east end of the building	40
Case 2-W			in the middle of the building	40
Case 3-W			at one end of the building	40
Case 4-W			at the two ends of the building	40
Case 4-20-W				20
Case 4-60-W				60
Case 4-80-W				80

**Table 9:** The relationship between the piloti ratio and the average wind velocity ratio.

Piloti Ratio (%)	Average Wind Velocity Ratio of Non-Piloti Area		Average Wind Velocity Ratio of Red Dashed Area	
	Summer	Winter	Summer	Winter
0	1.06	0.84	0.45	0.93
20	1.14	0.82	0.60	0.90
40	1.28	0.78	0.76	0.87
60	1.33	0.83	0.78	0.91
80	1.35	0.84	0.79	0.92

**Table 10:** The relationship between the piloti ratio and the average wind velocity.

Piloti Ratio (%)	Average Wind Velocity of Non-Piloti Area (m/s)		Average Wind Velocity of Red Dashed Area (m/s)	
	Summer	Winter	Summer	Winter
0	2.40	1.25	1.01	1.39
20	2.55	1.22	1.34	1.34
40	2.87	1.16	1.70	1.30
60	3.00	1.24	1.75	1.36
80	3.02	1.25	1.77	1.37
